# Evaluating Real-Time PCR to Quantify *Drosophila suzukii* Infestation of Fruit Crops

**DOI:** 10.3390/insects17010102

**Published:** 2026-01-16

**Authors:** Matthew G. Gullickson, Vincenzo Averello, Mary A. Rogers, William D. Hutchison, Adrian Hegeman

**Affiliations:** 1 Department of Horticultural Science, University of Minnesota, 1970 Folwell Ave, St. Paul, MN 55108, USAroge0168@umn.edu (M.A.R.);; 2 Department of Entomology, University of Minnesota, 1980 Folwell Ave, St. Paul, MN 55108, USA; hutch002@umn.edu

**Keywords:** invasive species, monitoring, spotted-wing drosophila, DNA, host plant resistance

## Abstract

Common methods for detecting *Drosophila suzukii* (spotted-wing drosophila, SWD) in fruit, such as microscopy, physical extraction, and incubation, are time-consuming and may underrepresent egg and first instar larvae counts, the smallest life stages of SWD. To address these limitations, we evaluated a quantitative real-time PCR (qPCR) protocol to detect and quantify SWD eggs using a linear model of the log-transformed ratio of eggs to sample volume (µL) in Tris buffer and fruit tissue. Compared to traditional approaches, this method reduces identification time from several weeks to approximately five hours. We observed a negative linear correlation between qPCR cycle threshold and egg concentration in both standard and fruit tissue samples, with similar model fits (R^2^ = 0.7215 for field fruit tissue; R^2^ = 0.874 for standard samples). This DNA-based protocol improves infestation detection speed and accuracy by enabling rapid, species-specific identification of *D. suzukii* in fruit tissue, addressing limitations of morphological identification of eggs and larvae. Further refinement for fruit tissue could enhance real-world applicability. Rapid detection may enable timely assessment of varietal resistance to SWD and support safer control strategies targeting early life stages, helping to prevent pest development and fruit degradation.

## Simple Summary

Early detection of invasive species helps growers know when to begin pest management, provides additional management options that target early life stages, and can reduce overall production losses. However, early detection of invasive insects is challenging due to their small size. Current methods for determining insect infestation in fruit rely on the time-consuming methods of either searching for eggs under a microscope or crushing fruit in a saline solution and counting the number of larvae that exit the fruit. These methods are also imprecise; definitive species identification requires rearing immature specimens to adulthood, by which time berries from the harvest may have already spoiled. To address these shortcomings, we evaluated a protocol for rapidly assessing the amount of spotted-wing drosophila (SWD) infestation in fruit using a DNA-based test, which measures the amount of SWD DNA in a fruit sample. We identified SWD with this protocol under laboratory settings and validated the protocol with fruit collected from the field. This test improves detection accuracy, enables quantification of the degree of SWD infestation, and reduces definitive species identification from several weeks to a few hours.

## 1. Introduction

Invasive insect pests threaten food and economic security [1]. A notable example is the recent and rapid spread of spotted-wing drosophila (*Drosophila suzukii*), an invasive vinegar fly, which was initially detected in North America in 2008 [2]. Since then, spotted-wing drosophila has colonized nearly all temperate fruit-producing regions across the globe [3,4,5,6] and devastated berry fruit production in its invaded range. There is currently no accepted threshold for spotted-wing drosophila-infested fruit, and distributors may refuse shipments of infested fruit [7]. An effective pest management program would ideally identify this pest rapidly, prior to shipment.

Current methods for detecting spotted-wing drosophila include baited traps for adults, larval extraction techniques such as floatation, dissection, or incubation, and egg counts via microscopy [8,9,10,11]. Although bait traps are useful for determining adult presence, they are imperfect due to high amounts of bycatch and poor correlation between the number of spotted-wing drosophila in the trap and fruit infestation [12,13], thus making larval and egg monitoring essential for assessing actual infestation rates. Common egg and larval monitoring methods, including microscopy, floatation, dissection, and incubation, are widely used by researchers and growers but vary in detection accuracy, require significant processing time for large samples, and can take up to two weeks when incubation or adult emergence methods are employed [10,11]. Furthermore, unless larvae are reared to adulthood, species identification may be inaccurate [14].

Molecular identification techniques, such as PCR, have been investigated for identifying spotted-wing drosophila adults and larvae [14,15,16,17,18]. For example, Zhai et al. [16] identified reference genes for reliable spotted-wing drosophila detection across multiple temperature treatments, life stages, and anatomical features using both conventional PCR and qPCR. Murphy et al. [17] developed markers for identifying spotted-wing drosophila within the *Drosophila* genus using multiplex PCR, which is more cost-effective than qPCR but does not provide quantitative data. Additionally, Dhami and Kumarasinghe [15] demonstrated the advantages of qPCR in achieving accurate and rapid identification of spotted-wing drosophila adults, particularly for degraded or low-DNA samples (e.g., eggs, legs) compared to conventional PCR. Despite these advancements, the methods in these studies still require extracting the insect pest from host tissue, which assumes prior knowledge of infestation.

Quantifying spotted-wing drosophila eggs directly within fruit tissue using qPCR remains unexplored but holds significant promise. Unlike other life stages, eggs represent the earliest point of infestation. However, fruit tissues present unique challenges for molecular identification assays because components such as phenols and polysaccharides can interfere with DNA extraction and amplification [19,20]. Establishing the accuracy of qPCR for detecting and quantifying insect eggs in fruit tissue could benefit researchers studying spotted-wing drosophila population dynamics, plant breeders evaluating resistant cultivars, and the fruit industry for post-harvest quality control. The objective of this study was to determine whether qPCR could detect and quantify insect infestation within fruit tissue by using spotted-wing drosophila and three host fruit as an example for its application in spotted-wing drosophila molecular identification. We hypothesized that there would be a negative linear relationship between the number of eggs per sample volume (µL) and qPCR cycle threshold (C_t_).

## 2. Materials and Methods

Spotted-wing drosophila used in this experiment were collected from primocane raspberries (*Rubus idaeus* ‘Caroline’) in St. Paul, MN, USA (45°0′31.09″ N, 93°18′56.313″ W), in September 2021 and reared in a growth chamber (16:8 L:D, 21 °C, 50% RH) on agar-yeast-sugar-cornmeal artificial diet [21]. Spotted-wing drosophila eggs were collected from each female’s ovipositor, and 100 eggs were placed in a 2 mL microcentrifuge tube and then homogenized in Tris (2-amino-2-hydroxymethyl-propane-1,3-diol, Sigma Chemical Company, St. Louis, MO, USA) buffer (100 µL) using a pellet pestle and stored at −20 °C. Tris buffer was prepared as a 1 M Tris base solution in deionized distilled water adjusted with Tris HCl to 8.0 pH at 23 °C measured using a pH electrode (MA850DIN electrode, Economy pH/EC Meter, Spectrum Technologies, Inc., Aurora, IL, USA). For all assays, DNA was extracted from spotted-wing drosophila eggs using a DNeasy Blood and Tissue Kit (Qiagen, Hilden, Germany). A ten-fold dilution series of spotted-wing drosophila eggs in Tris buffer was used to generate a standard curve with 4 subsamples (25 µL each) from each concentration. Standard egg concentrations were the equivalent of 1, 0.1, 0.01, and 0.001 eggs per µL of Tris buffer.

Store-bought blueberries (*Vaccinium corymbosum*), raspberries, and strawberries (*Fragaria* × *ananassa*) were artificially infested with spotted-wing drosophila by placing individual fruit in containers with three mated females and two males for 24 h. After briefly freezing to euthanize the adults, eggs were counted under a microscope. Field-grown, infested raspberries ‘Caroline’ and strawberries ‘Albion’ were harvested from organically managed research plots at the Minnesota Agricultural Experiment Station, St. Paul, MN, USA, on 8 July and 6 September 2024, respectively. Fruits were examined under a dissecting microscope to count *Drosophila* eggs per fruit, and fruit volume was recorded to calculate the egg concentration (spotted-wing drosophila eggs (#)/sample volume (µL)). Ten samples of each fruit species were selected to represent a wide range of egg counts (1 to 52 eggs) and sample volumes ranged from 3000 to 8500 μL. Each sample was homogenized for 1–2 min with a 7 × 195 mm probe on Power Gen 1000 Polytron (Fisher Scientific, Hampton, NH, USA) on setting #7 until all fruit tissues were visually homogeneous (i.e., no visible achenes, drupes, seeds, skins, or calyxes). Positive controls (spotted-wing drosophila eggs in Tris buffer) and negative controls (*Drosophila melanogaster* homogenate) were included. Two treatments, either with or without polyvinylpyrrolidone (PVP; 25 µL [22]), were tested for each fruit sample. Subsamples (25 µL) were combined with kit reagents in microcentrifuge tubes, vortexed, and incubated overnight at 56 °C. DNA extraction followed the kit instructions.

qPCR assays were performed using an iTaq SYBR Green Supermix 200 reaction kit (Bio-Rad Laboratories, Hercules, CA, USA) following instructions for 20 µL reactions. Spotted-wing drosophila-specific primers (Integrated DNA Technologies, Coralville, IA, USA) from cytochrome oxidase I genes were designed by Dhami and Kumarasinghe [15] (forward primer “ACTTGTGTCTTGTCCCTCACATAC” and reverse primer “GGCCCCAGATATAGCATTCC”) and had a melting temperature of 82.5–83 °C. Sample template DNA (6 µL) was added to individual wells of a 96-well qPCR plate (Bio-Rad Laboratories) with forward and reverse primers, SYBR green (10 µL), and sterile distilled water. The plate was sealed, centrifuged (2 min at 2000 rpm), and processed in a CFX96 Touch Real-time platform (Bio-Rad Laboratories). The qPCR settings were an initial 5 min increase to 95 °C, followed by 40 cycles of 5 s at 95 °C and 30 s at 60 °C, with fluorescence measured every cycle. The C_t_ threshold was set to 100 relative fluorescence units.

Data were analyzed in R version 4.3.2 [23]. The proportion of qPCR amplified samples for each treatment within each assay was calculated as a measurement of assay and treatment success. For each assay, linear models were fitted with the C_t_ value as the response variable predicted by log(eggs/µL). Model residuals were assessed visually to confirm they satisfied the assumptions of linear regression. Model fit was assessed based on the R-squared value of the linear model. Two-way ANOVA was used to determine differences among fruit species or PVP treatment.

## 3. Results

**Figure 1:**
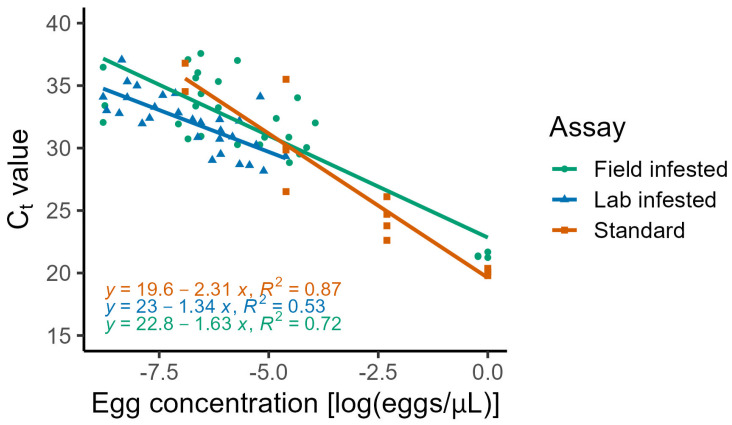
Assay comparison of quantitative real-time PCR cycle threshold (Ct) values in response to spotted-wing drosophila (*Drosophila suzukii*) egg concentration [log(# of eggs/sample volume (µL)] for the standard, lab-infested, and field-infested assays.

The proportion of samples that were amplified in the standard assay was 0.8125 ± 0.1008 (mean ± standard error of the mean (SEM)). There was a significant linear relationship between log transformed egg concentration and the C_t_ (F = 76.27, DF = 1, 11, *p* < 0.0001) and the R-squared value was 0.874 (Figure 1). The model equation was C_t_ = 19.622 + β_log(eggs/µL)_ × −2.308 ± 1.040.

The laboratory infested, store-bought fruit assay compared assay performance among blueberries, raspberries, and strawberries. Overall, 29 out of the 30 fruit samples were amplified (96.67%) during the qPCR assay; only one of the ten strawberry samples failed to amplify. Additionally, the positive control (spotted-wing drosophila eggs with Tris buffer (1 egg/µL, 25 µL)) was amplified, and the negative control (*D. melanogaster* with Tris buffer (1 fly/100 µL, 25 µL)) was not. There were no significant differences in amplification success rates among the three fruit species (F = 1.0, df = 2, 27, *p* = 0.381). There was a significant effect of log(eggs/µL) on C_t_ (F = 30.407, df = 1, 25, *p* < 0.0001), but there was not a significant effect of fruit species on the C_t_ (F = 0.9143, df = 2, 25, *p* = 0.4138), and therefore, the fruit species was removed from the model (Figure 1). The R-squared value for the model was 0.531. The regression equation from the fruit tissue assay was C_t_ = 22.998 + β_log(eggs/µL)_ × −1.340 ± 1.559.

The field-infested fruit assay success rate for raspberries without added PVP was 0.000 ± 0.000 and with PVP it was 1.000 ± 0.000. In the strawberries, the success rate without PVP was 0.700 ± 0.153, and with PVP was 0.800 ± 0.133. Fruit species had a significant effect on assay success (F = 6.081, df = 1, 36, *p* = 0.0186). The addition of the PVP treatment also had a significant effect on assay success (F = 29.432, df = 1, 36, *p* < 0.0001). Two-way ANOVA revealed that log(eggs/µL) significantly affected C_t_ (F = 143.08, df = 1, 24, *p* < 0.0001). Individually, fruit species influenced C_t_ (F = 7.1404, df = 2, 24, *p* = 0.004), and PVP treatment had a significant effect as well (F = 16.935, df = 1, 24, *p* = 0.0003). PVP treatment and fruit species interacted significantly (F = 19.703, df = 1, 36, *p* < 0.0001). The regression equation from the field-infested assay was C_t_ = 22.84 + β_log(eggs/µL)_ × −1.631 ± 2.543 and the R-squared value for this model was 0.7215 (Figure 1) [24].

## 4. Discussion

This qPCR protocol detected spotted-wing drosophila eggs both in Tris buffer solution and fruit tissue. As hypothesized, there was a negative linear relationship between the number of eggs per sample volume (µL) and qPCR C_t_. Quantifying this relationship enables not only the detection of infestation but also the future estimation of egg density based on Ct values. Our models have more variation in the data compared to another study which quantified spotted-wing drosophila DNA from traps which had an R-squared value of 0.9278 [18], but which did not have fruit tissue interfering with PCR reagents. Differences between the standard and lab assays with fruit tissue could have been due to components of the fruit tissue interfering with the DNA extraction and qPCR [19]. Assays with fruit tissue had flatter slopes compared to the standard assay that only contained spotted-wing drosophila eggs and Tris buffer, which indicates that the fruit tissue may have interfered with assay success. Berries contain high levels of polyphenols, which can degrade DNA polymerases, bind to DNA, and interfere with PCR by co-precipitating with nucleic acids. We attempted this assay with PVP to remove fruit phenols prior to DNA extraction. Adding PVP improved the proportion of samples that successfully amplified and reduced the C_t_ of our qPCR assays. Future research should investigate whether there is an optimal amount of PVP needed to remove enough phenols to maximize assay performance. Additional differences between the standard and lab assays may be due to the extent of homogenization between samples. Although fruit tissues were homogenized until visually homogeneous, this may still have not been enough to fully break up the eggs and equally distribute the DNA throughout the sample, potentially contributing to increased variance. Due to this challenge, we recommend future research into additional molecular identification techniques, such as using metabarcoding or environmental DNA methods for spotted-wing drosophila detection in fruit tissue.

Sample volume is a critical factor to consider when applying this assay. Large samples may prove challenging for scaling this protocol to commercial applications. Detecting one *Drosophila* sp. egg that is approximately 0.009 mm^3^ in volume and contains 16 cells within a large shipment of berries remains technically challenging even with molecular detection methods because this protocol still relies on destructive sampling and subsamples would need to be used for large shipments [25,26]. Spotted-wing drosophila larvae are larger and have more cells and therefore more copies of DNA than eggs. Consequently, the assay may be more sensitive to larvae compared to eggs; however, larval presence indicates that significant fruit damage has already occurred. Inadequate homogenization may leave eggs intact, increasing the risk that they are not represented in the 25 μL subsample used for analysis.

The protocol evaluated in this study demonstrates sufficient sensitivity to detect egg concentrations that we would observe in nature and has been validated with fruit infested in the field. Egg counts in the fruit tissue assays ranged from 1 to 52 eggs per sample, and sample volume ranged from 3000 to 8500 µL. These egg counts are comparable to what we have observed in previous studies based on direct count estimates [27,28]. Egg concentrations ranged from 4.7 × 10^−5^ to 1 (eggs/µL) and the log(egg concentration) ranged from −9.97 to 0. Therefore, this assay can detect spotted-wing drosophila eggs when there is approximately one or more eggs per strawberry fruit but may result in false negatives when there is fewer than one egg per strawberry, i.e., only one egg in a multiple berry sample. While the sample size for field-infested fruit (*n* = 10 per fruit type and treatment) was limited in this study, it demonstrates the feasibility of applying this qPCR under real-world conditions. We acknowledge that the small number of samples likely contributed to wider confidence intervals and reduced the precision of our regression estimates. This limitation means that the models should be interpreted as preliminary rather than broadly predictive. Future studies incorporating larger and more diverse datasets will be essential to refine these models and improve their generalizability across different fruit types and infestation scenarios.

## 5. Conclusions

The DNA-based identification protocol evaluated in this study contributes to enhanced speed and accuracy in detecting infestations. Further research to improve the method in fruit tissue would advance its real-world applicability. Use of qPCR technologies, with high sensitivity and specificity, could be utilized in the rapid assessment of spotted-wing drosophila or other invasive species infestations by pest quarantine organizations interested in minimizing the risk of new pest invasions to new countries or regions.

## Data Availability

The raw data supporting the conclusions of this article will be made available by the authors on request.

## Abbreviations

The following abbreviations are used in this manuscript:

qPCR	Quantitative real-time polymerase chain reaction
C_t_	Cycle threshold
PVP	Polyvinylpyrrolidone
